# Assessment of ecotoxicological effects of Fojo coal mine waste elutriate in aquatic species (Douro Coalfield, North Portugal)

**DOI:** 10.3389/ftox.2024.1334169

**Published:** 2024-02-23

**Authors:** Aracelis Narayan, Bárbara S. Diogo, Catarina Mansilha, Jorge Espinha Marques, Deolinda Flores, Sara C. Antunes

**Affiliations:** ^1^ Instituto de Ciências da Terra, Universidade do Porto, Porto, Portugal; ^2^ Departamento de Geociências, Ambiente e Ordenamento do Território, Faculdade de Ciências da Universidade do Porto (FCUP), Porto, Portugal; ^3^ Instituto Ciências Abel Salazar (ICBAS), Universidade do Porto, Porto, Portugal; ^4^ Departamento de Biologia da Faculdade de Ciências da Universidade do Porto (FCUP), Porto, Portugal; ^5^ Centro Interdisciplinar de Investigação Marinha e Ambiental (CIIMAR), Terminal de Cruzeiros do Porto de Leixões, Universidade do Porto, Matosinhos, Portugal; ^6^ Department of Environmental Health, National Institute of Health Doutor Ricardo Jorge, Porto, Portugal; ^7^ LAQV/REQUIMTE, University of Porto, Porto, Portugal

**Keywords:** coal mining, ecotoxicology, *Allivibrio fischeri*, *Lemna minor*, *Daphnia magna*

## Abstract

**Introduction:** The exploitation of anthracite A in the Pejão mining complex (Douro Coalfield, North Portugal) resulted in the formation of several coal waste piles without proper environmental control. In 2017, a new pedological zonation emerged in the Fojo area, after the ignition and self-burning of some of the coal waste piles, namely: unburned coal waste (UW); burned coal waste, and a cover layer (BW and CL, respectively); uphill soil (US); mixed burned coal waste (MBW); downhill soil (DS). This study aimed to evaluate the toxic effects of 25 soil elutriates from different pedological materials.

**Methods:** *Allivibrio fischeri* bioluminescence inhibition assay, *Lemna minor* growth inhibition assay, and *Daphnia magna* acute assay were used to assess the toxicity effects. Additionally, total chlorophyll and malondialdehyde (MDA) content and catalase (CAT) activity were also evaluated in *L. minor*.

**Results and Discussion:** The results obtained from each endpoint demonstrated the extremely heterogeneous nature of soil properties, and the species showed different sensibilities to soil elutriates, however, in general, the species showed the same sensitivity trend (*A. fischeri* > *L. minor* > *D. magna*). The potentially toxic elements (PTE) present in the soil elutriates (e.g., Al, Pb, Cd, Ni, Zn) affected significantly the species understudy. All elutriates revealed toxicity for *A. fischeri,* while US1 and UW5 were the most toxic for *L. minor* (growth inhibition and significant alterations in CAT activity) and *D. magna* (100% mortality). This study highlights the importance of studying soil aqueous phase toxicity since the mobilization and percolation of bioavailable PTE can cause environmental impacts on aquatic ecosystems and biota.

## 1 Introduction

Coal mining and its consumption negatively impact the natural environment (Ribeiro et al., 2010a; Li et al., 2022). Among several effects, the emission of harmful gases and particulate matter into the atmosphere, increased noise and waste generation around mining facilities, soil erosion and land use changes, acid mine drainage, water pollution, and potential impacts on local biodiversity (Fleeger et al., 2003; Stracher and Taylor, 2004; Ribeiro et al., 2010b; Ribeiro et al., 2011). Coal has been one of the dominant sources of energy over the last two centuries and provides 38% of the world´s electricity and 27% of global primary energy (WCA, 2022), however, coal mining waste (CMW) is a source of pollution to the environment (Wang et al., 2020). The knowledge of this material’s composition, origin, and characteristics help to understand the dynamics and the occurrence of potential toxic elements (PTE) in different environmental matrices (Stefaniak and Twardowska, 2010). The disposal of CMW and the possible occurrence of self-combustion represent an increased risk to the environment and human health due to the release of organic compounds (e.g., polycyclic aromatic hydrocarbons), the leaching of PTE, and the possibility of bioaccumulation and biomagnification in the trophic webs (Stefaniak and Twardowska, 2010; Jafarabadi et al., 2018). Nevertheless, the ecosystem, specially soils, appears to be an excellent filter since it has the ability to retain contaminants such as PTE. However, pollutants can be transferred into the soil, groundwater, and surface water, either in dissolved or particulate form (Querol et al., 2008; Izquierdo and Querol, 2012; Oliveira et al., 2016). Moreover, the storage of coal waste in disposal piles can cause potential problems for terrestrials, namely the aquatic biota that may be subjected to runoff and leachate from these piles (Hall et al., 1982).

Mining activities promote a large environmental modification in the landscape’s outline, chemistry, and biology (Sonter et al., 2018). The acidic nature of water circulating in coal mine waste usually results from the oxidation of pyrite, and the low pH increases the dissolution of PTE, favoring their transport in the leachates (Costa and Duarte, 2005; Nyström et al., 2021). The chemical mobility of pollutants is fundamental to understand toxicity, bioavailability, and geochemical behavior (Dang et al., 2002; Ribeiro et al., 2010a). Indeed, a wide range of significant effects such as an increase in morbidity and mortality, a reduction of the number of plant and animal species, reductions in plant growth, and loss of visual aesthetic landscape characteristics can result from this phenomenon (Cehlár et al., 2016).

Then again, ecotoxicological tools have been proven to be a sensitive and appropriate approach for assessing toxicity and estimating the environmental risk of contaminants (Alvarenga et al., 2008) since chemical methods need to be complemented with a biological procedure to explain the effects on the ecosystem’s biota (Lee et al., 2015; Abd Aziz et al., 2019). However, research on toxicology in coal mines and CMW is scarce, and the impact of PTE-enriched effluents on terrestrial and aquatic biota is poorly known (Martin and Black, 1998; Shylla et al., 2021).

Several authors have described that standardized assays are sensitive to assess the environmental risk assessment of leachate and elutriate of mining wastes using different model species (e.g., bacteria, invertebrates, macrophytes) (e.g., Sackey et al., 2020). Antunes et al. (2007) considered the behavioral response of *Eisenia andrei* (model species in soil ecotoxicology) a sensitive endpoint since it allowed the evaluation of the toxicity of soils from areas subjected to runoffs from uranium mines. The same authors, Antunes et al. (2007) assessed the toxicity of sediment elutriates from artificial ponds from a uranium mine exploitation (with low pH and high concentration of PTE) using acute assays with *D. magna* and *D. longispina* (EC_50_ = 96.3% and 94.7%, respectively). Gauthier-Manuel et al. (2021) reported that after exposure to acid drainage from a coal mine, a growth inhibition and an emergence delay of model organism *Chironomus riparius* larvae were observed. On the other hand, biochemical biomarkers are frequently used to assess the effects of organisms’ exposure to environmental contaminants (Lionetto et al., 2019). Biomarkers are a measurement of physiological changes (e.g., pheopigments) in cells or tissues and serve as indicators of the presence and/or effects of environmental contamination (Mouneyrac and Amiard-Triquet, 2013). Biomarkers also examine whether normal detoxification (e.g., oxidative stress) or repair capacities have been exceeded. Catalase (CAT) represents one of the first enzymes involved in the antioxidant defense system (responsible for the catalysis of hydrogen peroxide, a reactive oxygen species that if not eliminated or neutralized by antioxidant defenses can induce oxidative stress, in water) (Alkimin et al., 2020). On the other hand, malondialdehyde (MDA) is a final product of lipid peroxidation, that can be used as an important indicator of physiological stress (Diogo et al., 2023).

Previous studies on the assessment of the environmental impact of coal mining waste in the Douro Coalfield (North of Portugal) have mainly focused on geochemical analysis and characterization (Ribeiro et al., 2010a; Ribeiro et al. 2011; Ribeiro et al. 2020; Ribeiro and Flores, 2021), leaving room to an ecotoxicology-based environmental risk assessment. The present research adopted an ecotoxicological approach and the main goal was to assess the potential toxicity of soil elutriates from coal mining wastes (burned and unburned areas) in the aquatic ecosystem. To achieve this goal, a set of bioassays was conducted with aquatic model species from different trophic levels, and several endpoints were evaluated: Microtox assays—bioluminescence inhibition of the bacteria *Aliivibrio fischeri*, Acute assays—mortality of *D. magna*, and growth inhibition of *L. minor*. Additionally, sub-individual parameters were also evaluated in *L. minor* [total chlorophyll content, catalase (CAT) activity, and malondialdehyde (MDA) content] after exposure to the different soil elutriates.

## 2 Materials and methods

### 2.1 Study site

**FIGURE 1:**
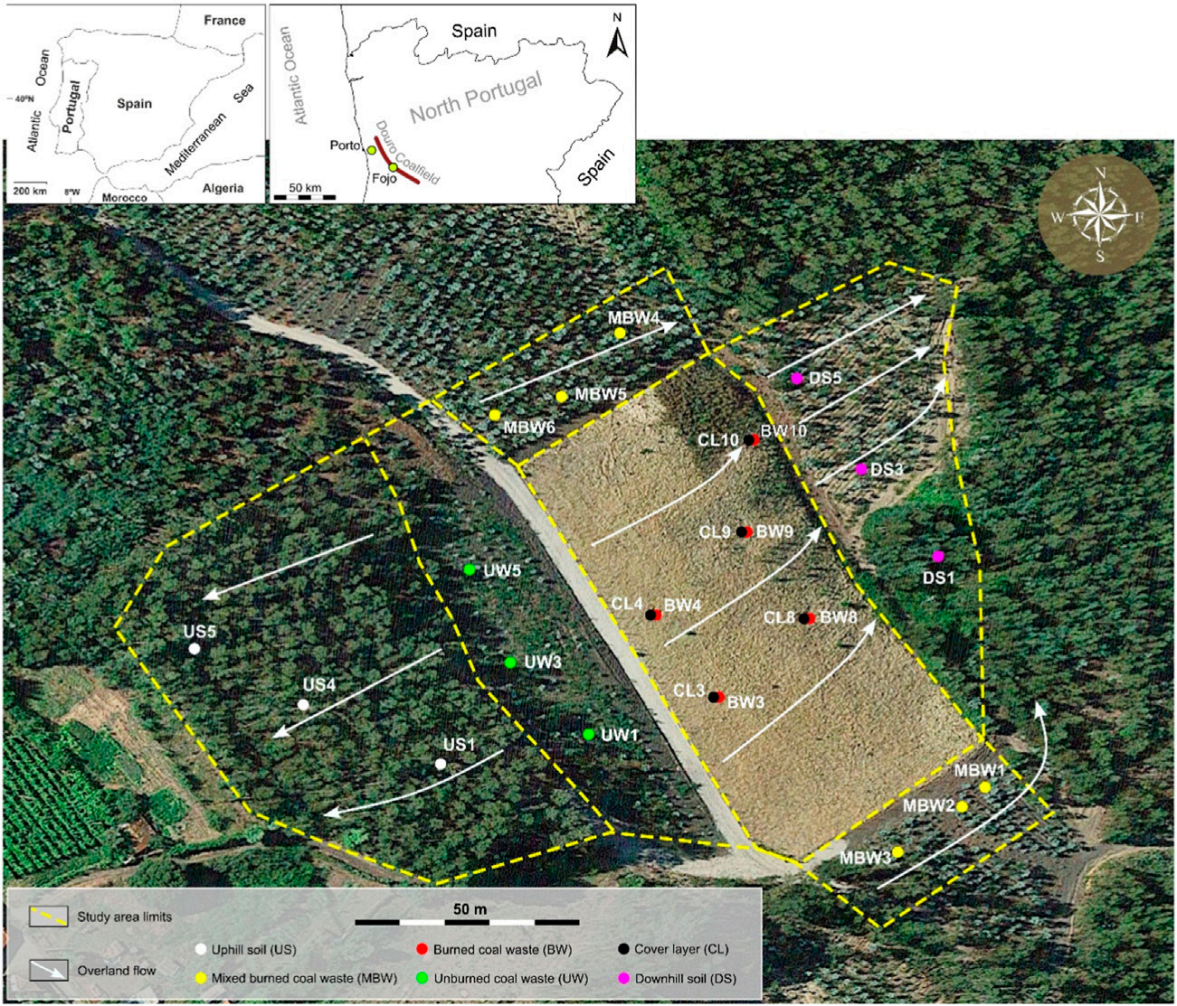
Fojo mine coal waste disposal area and pedological zonation, with the location of the sampling sites.

The Fojo mine is located in the northeast of Portugal, in Pedorido, Castelo de Paiva (Figure 1). This mine is one of the principal mining areas included in the Pejão region of Douro Coalfield, and coal was extracted from 1920 until 1994, where the greater production was from 1940 to 1968 (Machado and Cabral, 1970; Caetano, 1998; Costa, 2021). The Fojo waste piles started burning in the summer of 2017 due to a wildfire that occurred in the area. For 2 years (between 2017 and 2019) an effort to extinct the fire was carried out, and to stop the combustion process the remobilization of the coal-waste material and a mixture of water with cooling accelerator agent was used. The occurrence of self-burning and the intervention for its extinction resulted in a new pedological zonation (Figure 1), specifically: i) Uphill soil (US) – soil without mining influence, with Regosol/Cambisol features; ii) Unburned coal waste (UW)—without self-burning, with Technosol features; iii) Burned coal waste (BW)—with self-burning coal waste material and Technosol features; iv) Protective cover of the burned coal waste (CL) – 30 cm–40 cm thick cover layer made of geological material from another site deposited on top of BW after the extinction of self-burning; with Technosol features; v) Mixed burned coal waste (MBW) – composed of a mixture of burnt or unburnt material, as a result of the remobilization process of the waste material during the intervention to extinguish self-burning and to the subsequent tillage for eucalyptus plantation; with Technosol features; vi) Downhill soil (DW)—situated downhill from the BW material; with Regosol/Cambisol/Anthrosol features.

### 2.2 Sampling and general soil physical and chemical characterization

The present study was conducted with 25 soil samples, distributed according to the previously described pedological zones (see description in Section 2.1 – Figure 1). Soil samples [from the upper 20 cm of: i) A horizon, in US (US1, US4, US5) and DS (DS1, DS3, DS5); ii) the most superficial layer of UW (UW1, UW3, UW5), MBW (MBW1, MBW2, MBW3, MBW4, MBW5, MBW6), and CL (CL3, CL4, CL8, CL9, CL10); iii) covered BW (BW3, BW4, BW8, BW9, BW10)] were collected and stored in opaque plastic bags and stored at 4°C, in the laboratory, until analyses were performed. For soil physical and chemical determinations (pH, conductivity, and water holding capacity) samples were air-dried for 1 week, homogenized, and sieved through a 4 mm mesh.

Soil pH and conductivity were measured in a soil-water suspension (extraction ratio 1:5 w/v) according to the method described in FAOUN (1984). Each replicate of soil (10 g) was mechanically shaken with 50 mL of deionized water for 15 min. After the mixtures had stood for 1 h, the solution pH was measured with a multiparametric probe (Multi 3630 IDS SET F). The soil-water suspension was left to rest overnight, and on the following day, the conductivity of the samples was measured with a multiparametric probe (FAOUN, 1984).

Water holding capacity (WHC) was determined in each soil, where the soil was placed in plastic bottles (where the background was replaced by filter paper) and immersed in water for 3 h (ISO, 2008). After this period, the samples were drained for 2 h by rejecting excess water with absorbent paper. The WHC was determined by weighing each replicate before and after drying at 105°C until the weight stabilized (ISO, 2008).

To leach the heterogeneous soil sample for metals and nutrients bioavailable in the aqueous solution, the USGS Field Leach Test (Hageman, 2007) was used. Bicarbonate (HCO_3_^−^), and oxidability were analyzed by titration. The major inorganic ions (Na^+^, K^+^, Mg^2+^, Ca^2+^, Li^+^, Cl^−^, NO_3_^−^, F^−^, and SO_4_^2−^) were analyzed by ion chromatography, CI (DionexTM system DX-120/ICS-1000, Dionex Corporation, Sunnyvale, CA, USA), and PTE (Cr, Mn, Ni, Cu, Zn, As, Cd, and Pb) in a Varian AA240 Atomic Absorption Spectrometer (Varian Inc., Palo Alto, CA, USA). Other components, such as Al, Fe, NO_2_^−^, and NH_4_^+^, were analyzed in a Continuous Segmented Flow Instrument (CSF) (San-Plus Skalar, Skalar Analytical, Breda, The Netherlands).

### 2.3 Elutriate assays

Elutriates were prepared using a ratio of 1:4 (w/v) (USEPA, 1998) of each soil sample with a respective culture medium of each species: distilled water for Microtox® assay (Microbics, 1992), ASTM hard-water medium for *D. magna* assay (ASTM, 1989; OECD, 2004), and Steinberg medium for *L. minor* assay (OECD, 2006). The soil suspension was mechanically stirred (in an orbital mechanical shaker, 100 rpm) for 12 h at room temperature, followed by a period deposition of 12 h. After this period the elutriate was collected by decantation. All the assays were performed using the direct elutriate - without dilution and used in the following 48 h.

#### 2.3.1 *Aliivibrio fischeri*—Microtox® bioluminescence inhibition assay

Standard Microtox® assays were performed using liquid phase (elutriates) procedures according to standardized protocols (Microbics, 1992). Lyophilized luminescent marine bacteria *A. fischeri* (NRRL strain number B-11177) was used for this assay, after rehydration with a reconstitution solution before starting the assay. Bioluminescence evaluation was performed 5 min after exposure, at 9 dilutions of each sample (dilution factor of 2), using the Microtox Model 500 toxicity system with an automatic luminescence recording, following the protocol of the Microtox^®^ Acute Toxicity Basic Test Procedures manual—Modern Water. EC_20_ values were used to report toxicity values for the elutriates.

#### 2.3.2 *Daphnia magna* – acute assay

*Daphnia magna* acute assay was performed according to the standard guideline OECD 202 (OECD, 2004) with six-well microplates adaptation. Each elutriate was assessed with four replicates, each one containing 12.5 mL of elutriate, and five neonates (less than 24 h old born between the 3rd and the 5th broods). A negative control was conducted with *D. magna* neonates exposed to ASTM medium. The assay was performed under controlled conditions of temperature (20 ± 2°C) and photoperiod (16 h^L^:8 h^D^). After 48 h of elutriate exposure, the organisms were observed, and dead organisms were counted to assess the percentage of effects induced by each elutriate. According to OECD guideline nº 202 (OECD, 2004), dead organisms (mortality) are considered when organisms present total absence of movements of the thoracic appendages and heartbeats. The interpretation of results was made according to mortality.

#### 2.3.3 *Lemna minor*—growth inhibition assay

*Lemna minor* growth inhibition assays were conducted according to standard guidelines (OECD, 2006), with microplate adaptation (Diogo et al., 2023). Four replicates per elutriate were used, and in each replicate 12.5 mL of elutriate and four fronds of *L. minor* were added. The control group was conducted with fronds exposed to the Steinberg medium. The assays were conducted for 7 days, and the microplates were maintained under controlled conditions of light (24 h, ∼7000 lux), and temperature (23 ± 2°C). At the final of the exposure period (7 days), the number of fronds was counted, and the results were expressed in the percentage of growth inhibition (OECD, 2006). Afterward, the fronds were washed with distilled water, dried with absorbent paper, weighted, and stored in Eppendorf microtubes at −80°C for quantification of the total chlorophyll content and biochemical determinations [catalase (CAT) activity and malondialdehyde (MDA) content] (three replicates for each parameter).

The total chlorophyll content was quantified according to the method defined by Lichtenthaler (1987). Pigments extraction occurred in 1 mL of 96% ethanol at 4°C overnight. After this period the absorbance was measured through spectrophotometry at the wavelengths of 644 and 649 nm (GenesysTM 10Series Thermo Spectronic). For the CAT enzymatic determinations, samples (∼5 mg of fronds per replicate) were sonicated in 1 mL of ice-cold phosphate buffer (50 mM, pH 7.0) with 0.1% Triton X-100. Catalase is an antioxidant defense enzyme, responsible for the decomposition of hydrogen peroxide into water and molecular oxygen (Alkimin et al., 2020) and was quantified according to Aebi (1984), adapted to a 96-well plate. CAT activity was expressed as millimoles of H2O2 consumed per minute, per mg fresh weight. For MDA quantifications, biological samples (∼5 mg of fronds per replicate) were sonicated in 500 µL of 0.1% trichloroacetic acid. Its content was determined by the thiobarbituric acid method as described by Elkahoui et al. (2005) and expressed as μM MDA equivalents per mg fresh weight. The specific and the non-specific absorbance of the supernatant were measured at 532 and 600 nm, respectively. The concentration of MDA was calculated by subtracting the non-specific absorbance and using the molar extinction coefficient ε = 155 mM/cm.

### 2.4 Statistical analysis

Data of soil samples based on soil physical, chemical properties, and PTE from the elutriate, were used to conduct a Principal Component Analysis (PCA) to perceive the chemical characterization of each soil elutriate. For ecotoxicological assay results, Pearson correlation analyses were conducted to discriminate each chemical elutriate parameter responsible for biological effects. All the evaluated endpoints (pigments and biomarkers results) were checked for normality by the Shapiro-Wilk test and for homogeneity of variances by Levene’s test to verify compliance with the ANOVA assumptions. One-way ANOVA was used in the pigments and biomarkers results, followed by a *post hoc* Dunnett test to discriminate significant differences between elutriate samples and the control treatment. Statistical analysis was performed with SPSS Statistics v29 and the adopted level of significance was 0.05.

## 3 Results and discussion

### 3.1 Physical and chemical parameters and elutriate PTE

**FIGURE 2:**
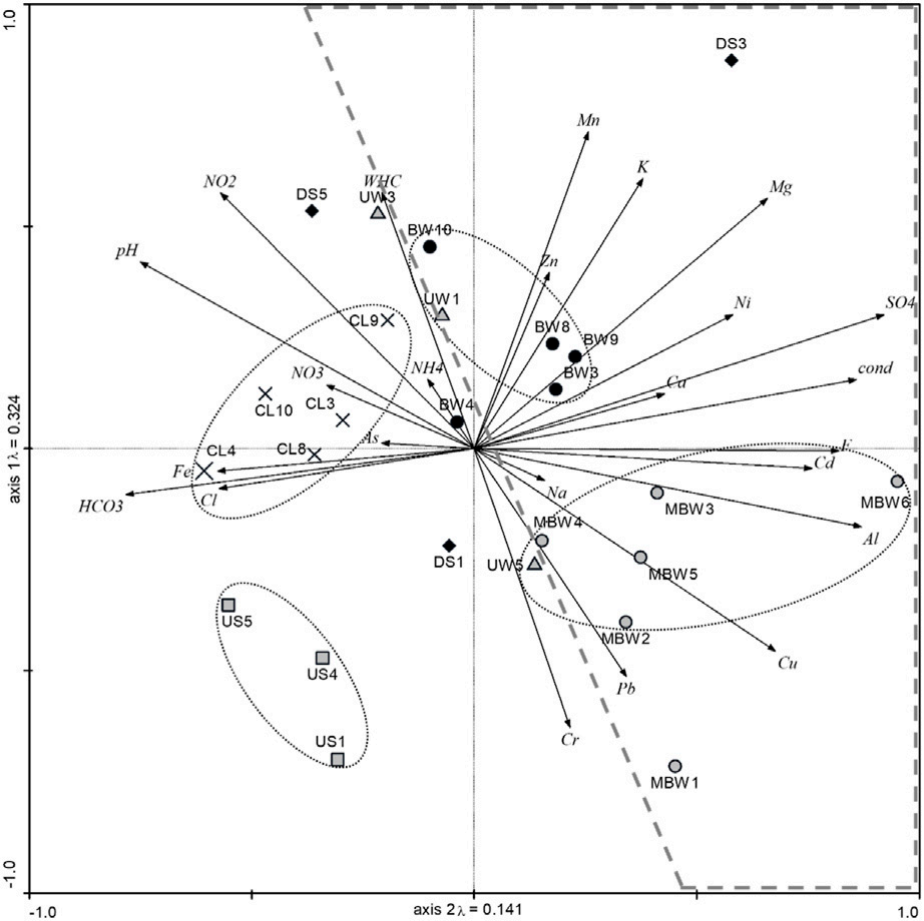
PCA result of physical and chemical parameters (for parameters abbreviations see Section 2.2) measured in soil elutriates. Soil sampling areas are evidenced by oval dotted lines, and burned areas are all included inside the dashed line.

In the study area, soils composed of technogenic materials (coal mine wastes) are classified as Technosols (FAO, 2015; Espinha Marques et al., 2021). Supplementary Table S1 presents the concentration of nutrients and trace metals recorded in the soil elutriates, and Figure 2 shows the PCA results regarding the matrix of physical and chemical parameters measured in each soil elutriate. The PCA analysis indicates a clear separation between soils from burned (dash line) and unburned coal wastes. The soil samples of each site seem to be replicates due to the closeness recorded (oval dotted lines). On the other hand, the unburned coal waste samples (UW) and downhill soil samples (DS) showed individual behavior (appear not clustered). PCA analysis also indicates that the uphill soils (US) do not show any relation with the physical and chemical parameters measured, while the cover layer (CL) and unburned coal waste (UW) were characterized by high values of WHC, NH_4_^+^, NO_2_^−^, NO_3_^−^, As, and Fe. Higher concentrations of trace elements (Zn, Ni, Cd, Cu, Pb, and Cr), soluble cations (K, Na, Mg, Ca), macronutrients (Al, Mn), inorganic ions (SO_4_^2−^, F^−^) appear associated with the burned coal wastes (MBW and BW, Figure 2), which is in line with other studies that report that elements leaching can be temperature-dependent (Espinha Marques et al., 2021; Ribeiro and Flores, 2021).

Soil electric conductivity (EC) ranged between 33 and 366 μS/cm. The lowest EC values were observed in soil US5 and CL4, and the highest in UW5, MBW6, and DS3 soils. However, DS3 and DS1 (downhill soils) have very high EC values 366 μS/cm and 168 μS/cm respectively, compared with US soils (uphill soils). This fact could demonstrate that the leachates from burned and non-burned piles, may contribute to the enrichment of ions bioavailability. Despite that, none of the Fojo coal waste samples’ EC values overcome the reference value limits for EC (475 μS/cm) proposed by the Portuguese Environment Agency for agricultural soils (APA, 2019). Moreover, compared with values detected in other coal waste piles from North of Portugal, the here-obtained results were lower than Serrinha mine (86.9–1,410 μS/cm) (Ribeiro et al., 2010b), and similar to São Pedro da Cova mine (22–475 μS/cm) (Santos et al., 2023).

Soil water holding capacity (WHC) defines the ability of soil to hold water at a particular time (Adhikari et al., 2022). The studied samples showed a lower WHC for US soils and the highest values for CL samples. Regarding the remaining samples, the WHC was similar, ranging between 23% and 35.8%. Increased soil WHC is mostly associated with higher infiltration rates and lower runoff thus it could decrease the potential for soil erosion. Soil with low WHC loses a significant portion of rainwater by deep percolation, leaching nutrients from the root zone, leading to inefficient use of resources and adverse environmental problems (AbdAllah et al., 2021). The burned coal waste samples (BW) and mixed burned coal waste (MBW) correspond to soils in which pollutants are more leachable and potentially transported to downhill soils (DS).

According to the measured pH, all soils are acidic. The lowest pH value was measured in UW5 (pH = 3.5), and the highest in US5 (pH = 5.15). Ribeiro and Flores (2021) suggested, for similar areas in the Douro Coalfield, that this pH range could be promoting significant acid mine drainage (AMD) of PTE. It is necessary to understand the mineralogy of the coal residues that incentive AMD production by mineral as pyrite and jarosite and the concentration of the minerals that work as neutralizers: calcite, aragonite, and siderite (Çelebi and Ribeiro, 2023). The release of PTE will depend on the exposure leaching time and the pH (Li et al., 2022; Liu et al., 2023).

### 3.2 Bioassays

#### 3.2.1 *Allivibrio fischeri*

Table 1 shows the EC_20_ values for bioluminescence inhibition of the bacteria *Allivibrio fischeri* after exposure to the soil elutriates. All the elutriates reveal toxicity for *A. fischeri*, and CL8 (EC_20_ = 0.88%) was the most toxic, while CL4 was the least toxic (EC_20_ = 55.9%). For CL8 soil elutriate acid pH values were recorded (pH = 4.78) as well as high values of HCO_3_ and Fe. Berglind et al. (2010) already showed that light production was highly pH dependent and concluded that the optimal pH for the growth of *A. fischeri* is 6.8 (higher than recorded in CL8 elutriate), and below this value, the luminescence rate was significantly lower (Silva et al., 2021). In addition, high concentrations of Fe > 3.1 mg/L affected the stability of the bioluminescence capacity of this species (Futra et al., 2014; Teixeira et al., 2014). Contrarly, Haygood and Nealson (1985) reported that extremely low concentration of Fe (0.16 mg/L) also inhibited this bacteria luminescence.

**TABLE 1:** Results of acute effects for the bioassays performed with *Allivibrio fischeri*, *Lemna minor*, and *Daphnia magna* after exposure to the different soil elutriates.

Soil Samples	*A. fischeri* EC_20_ (%)	*L. minor* Growth inhibition (%)	*D. magna* Acute effects (%)
US1	8.70	61.8 ± 6.6	100 ± 0.0
US4	2.48	47.2 ± 4.9	70.0 ± 12.9
US5	8.72	55.0 ± 5.2	5.00 ± 5.0
UW1	7.81	8.30 ± 3.8	10.0 ± 5.8
UW3	55.8	−0.10 ± 4.0	0.00 ± 0.0
UW5	2.19	85.0 ± 4.1	100 ± 0.0
MBW1	13.2	−3.20 ± 3.0	0.00 ± 0.0
MBW2	3.37	27.5 ± 1.3	75.0 ± 25.0
MBW3	15.4	31.5 ± 6.6	20.0 ± 8.2
MBW4	12.8	14.2 ± 3.7	0.00 ± 0.0
MBW5	5.41	27.8 ± 3.8	20.0 ± 8.2
MBW6	3.39	32.3 ± 4.2	15.0 ± 5.0
BW3	6.80	42.7 ± 8.0	0.00 ± 0.0
BW4	1.42	16.6 ± 2.7	0.00 ± 0.0
BW8	9.93	16.2 ± 3.2	0.00 ± 0.0
BW9	1.97	9.20 ± 3.6	5.00 ± 5.0
BW10	1.51	9.10 ± 4.0	0.00 ± 0.0
CL3	1.53	3.10 ± 3.7	0.00 ± 0.0
CL4	55.9	7.20 ± 2.7	0.00 ± 0.0
CL8	0.88	4.50 ± 2.2	0.00 ± 0.0
CL9	8.04	−4.50 ± 1.6	0.00 ± 0.0
CL10	1.61	7.70 ± 2.8	0.00 ± 0.0
DS1	4.99	6.90 ± 6.6	5.00 ± 5.0
DS3	6.02	4.00 ± 7.8	15.0 ± 15.0
DS5	3.69	7.80 ± 3.4	0.00 ± 0.0

EC_20_ toxicity endpoint showed a positive and significant correlation with macronutrient Fe (*r* = 0.544, *p* = 0.005), inorganic ion NO_2_^-^ (*r* = 0.447, *p* = 0.025), and pH (*r* = 0.443; *p* = 0.027). Indeed, Scheerer et al. (2006) already showed that the bioluminescence of *A. fischeri* could be affected by factors such as temperature, pH, ionic strength, nitrogen, and carbon source. In the here-present study, and according to physical and chemical results, *A. fischeri* bioluminescence seems to be affected by high values of pH, WHC, and NH4^+^, parameters that characterize the CL elutriates (Supplementary Table S1).

#### 3.2.2 *Daphnia magna*

*Daphnia magna* acute toxicity results are shown in Table 1. Elutriates from soils US1 and UW5 proved to be the most toxic, causing 100% mortality of *D. magna* in less than 24 h. Elutriates from soils US4 and MBW2 caused more than 70% of the mortality, while the remaining soil elutriates induced less than 20% *D. magna* mortality. A negative and significant correlation between *D. magna* and pH (r = −0.416; *p* = 0.039), WHC (*r* = −0.620; *p* < 0.001), and K (*r* = −0.442; *p* = 0.027), and a positive correlation with OM (*r* = 0.437; *p* = 0.029) was observed. These results could be explained by the acid pH or/and concentration of PTE recorded in the soil elutriates (Supplementary Table S1). On the other hand, OM may influence *D. magna* toxicity namely with metals combination (e.g., Fan et al., 2016). Overall, for this species burned coal wastes (BW, MBW) seem to be less toxic than the unburned soils (US, CL, DS), this could be explained by the higher OM content and the potential association with metals (Adusei-Gyamfi et al., 2019). Indeed there is a competition of cations for binding sites in OM with preference of cations depending on the organic radical, and pH values (Adusei-Gyamfi et al., 2019). Moreover, soils with lower WHC seem to increase toxicity to *D. magna* since this type of soils with lower WHC will not be able to retain nutrients and metals will be bioavaliable in elutriates (AbdAllah et al., 2021). US soils should serve as potential reference soils since they have not been exposed to the impact of coal mining, trace metals concentration are lower than the burned and unburned coal waste (Supplementary Table S1). However, the results indicate that the US soils have physical, and chemical properties, and types of vegetation (e.g., *Pinus pinaster*, *Arbutus unedo*, *Eucalyptus*, and Bryophyta), which can confer them high acidity.

Several studies already demonstrated that a mixture of chemical component with changes in pH, OM, WHC, and K values could have an influence and may induce acute toxicity to *D. magna* (e.g., De Schamphelaere and Janssen, 2002; Loureiro et al., 2005; Yim et al., 2006; Alvarenga et al., 2008; Maisto et al., 2011; Pukalchick et al., 2017). The UW5 elutriate showed a high concentration of metallic elements (Al, and Pb), while MBW2 showed high concentrations of Cr and Ni. Some studies suggested that Cd and Cu showed more toxicity to *D. magna* than Pb and Cr (Khangarot and Ray, 1989; Okamoto et al., 2015), depending on the concentration of PTE and the time of exposure (Peng et al., 2022). Aluminum seems to be a toxic element for *D. magna*. However, the concentration of Al recorded in the UW5 and US1 (more toxic soil elutriates; 0.199 mg/L and 0.039 mg/L, respectively) was lower, than the EC_50_ reported by the literature [EC_50_ (48 h) = 0.586 mg/L, at 5.10–6.40 pH] (Fikirdeşici-Ergen et al., 2018). To ensure protection against acute toxicity to aquatic species, vertebrates, and invertebrates, the United State Environmental Protection Agency (USEPA, 1998) criteria recommend that the average concentration of aluminum in water samples, must not exceed 0.087 mg/L more than once every 3 years when pH is between 6.5 and 9. However, in the present study, several elutriates (e.g., UW5, MBW, BW, CL9, DS1, and DS3) exceed this reference value (e.g., the maximum concentration detected was 0.963 mg/L in the MBW6 elutriate). Despite the concentration of Al reported, the high toxicity can be the result of the complex mixture of PTE detected in soil elutriates (Peng et al., 2022), but also by the low pH, and low water hardness (given by Ca and Mg ions) recorded in the elutriates. Furthermore, Pb and Cr showed toxicity for *D. magna* at 0.10 mg/L Pb and 0.12 mg/L Cr, however, when they were in a metal mixture, an increase of toxicity was observed (0.062 mg/L for Pb and 0.015 mg/L for Cr) (Jeong et al., 2001; Mansour et al., 2015). Other studies also report *Daphnia magna* acute toxicity for Ni and Zn at 0.1–1 mg/L and for Cd and Cu at <0.1 mg/L (e.g., Okamoto et al., 2015). Meyer et al. (2015) reported a *D. magna* EC_50_ value for Cu of 0.103 mg/L.

#### 3.2.3 *Lemna minor*

*Lemna minor* is recognized as an important bioindicator with ecological relevance for monitoring different pollution sources (Garnczarska and Ratajczak, 2000). The results obtained in the *L. minor* growth inhibition assay after exposure to soil elutriates are shown in Table 1. The highest growth inhibition (>85%) was observed when *L. minor* was exposed to UW5. This soil elutriate showed an acid pH and higher EC, and metals (Al, and Pb). The growth inhibition can be associated with the low pH (3.5) recorded, since the optimal pH value for *L. minor* growth is from 6 to 8 (Chong et al., 2003; Kaur et al., 2012; Iqbal and Baig, 2016). In addition, the concentration of Al is commonly considered an abiotic stress factor under acidic conditions (Su et al., 2019), and it is a potential growth-limiting factor for plant development in acidic soils (Rout et al., 2001). It has already been reported that 0.196 mg/L of Al causes a toxic effect in *L. minor* after 48 h of exposure (Fikirdeşici-Ergen et al., 2018). The authors showed that when Al is in solution, it hydrolyzes and, with a low pH, the increase of H^+^ causes damage to the species and contributes to mortality (Satizábal et al., 1999). Su et al. (2019) showed that Al is toxic for root growth, which interferes with different mechanisms, including cell elongation and division, competition with K, Ca, Mn, Cu, and Mg for binding sites, disrupting a variety of biological process and oxidative stress pathways. *L. minor* was also highly affected after exposure to US1, US4, US5, and BW3 (growth inhibition between 40% and 60%). Indeed, a significant and negative correlation with *L. minor* growth inhibition was recorded with pH (r = −0.504; *p* = 0.010), WHC (*r* = −0.672; *p* < 0.001), and K (*r* = −0.462; *p* = 0.020). Low concentration of K in plants, reduces photosynthetic CO_2_ fixation followed by membrane and chlorophyll damage, thus alterations of leaf anatomy, decreases in the chloroplast surface area and the content of chlorophyll and increase antioxidants activities (Johnson et al., 2022). Radić et al. (2010) studied the ecotoxicological assessment of industrial effluent using *L. minor* and showed that metals are toxic (Zn>Ni>Fe>Cu>Cr>Pb) for *L. minor* tissues with different ranges of damage (e.g., Pb exposure increase proline content, and cause inhibition of *L. minor* growth). Dirilgen (2011) studied the effects of Pb on *L. minor* growth inhibition, during 4 and 7 days, and the EC_50_ values for Pb were estimated as 6.8 to 5.5 mg/L (higher values than those detected in soil elutriates in the present study such as for MBW2 = 0.004 mg/L). Van Dyck et al. (2023) studied the effects of Mn and recorded a decrease of *L. minor* growth at concentrations above 70 mg/L, higher values than observed in the here-elutriates (Supplementary Table S1). Moreover, *L. minor* showed growth reduction in As presence above 2 mg/L (Duester et al., 2011), value also up to the here-present study (Supplementary Table S1). Trace elements such as Cd cause visible damage to *L. minor* at 0.5 mg/L (Khellaf and Zerdaoui 2009), once again values up to the observed in our elutriates (Supplementary Table S1). On the other hand, several studies showed low toxicity for *L. minor* after exposure to some metals, namely Mn at 25 mg/L (Van Dyck et al., 2023), Fe at 22.6 mg/L (Teixeira et al., 2014), Zn 15 mg/L (Van Dyck et al., 2023), Ni at 2.5 mg/L (Nogueira et al., 2020) where no significant effects in the growth rate were recorded. Despite the low metal toxicity for *L. minor*, mentioned above, the physical and chemical parameters such as pH, hardness, and the presence of several metals may have contributed to the here-observed effects.

**FIGURE 3:**
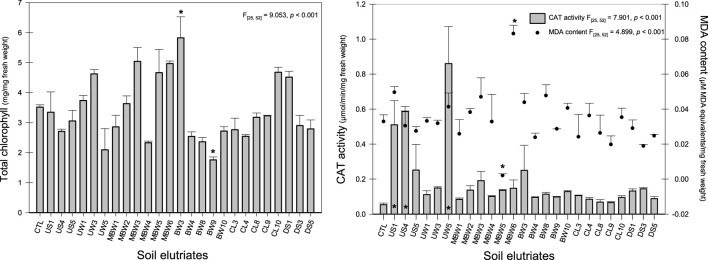
Results of *Lemna minor* pigments (total chlorophyll content) and biochemical biomarkers [Catalase (CAT) activity and Malondialdeyde (MDA) content] after exposure to the different soil elutriates. Data are expressed as mean ± standard error; * Stands for discriminate differences between CTL group and soil elutriates (Dunnett test, *p* < 0.05).

Total chlorophyll and biomarkers (CAT activity, and MDA content) of *L. minor* have also been used to assess the toxicity of soil elutriates (Figure 3). A significant increase in the concentration of total chlorophyll was recorded for *L. minor* after exposure to BW3 soil elutriate, while a significant decrease was observed in BW9 soil elutriate (both burned coal wastes). Indeed, both elutriates showed a similar concentration of Mg, Al, Ni, Mn, and Zn. For BW3 elutriate the increase in chlorophyll content could be explained due to the possibility that PTE exposure can induce a response in plants, leading to an increase in chlorophyll (Chandra and Kang, 2016). This response is often a defense mechanism to counteract the stress and maintain photosynthetic activity. Chandra and Kang (2016) studied mixed heavy metal (Cd, Cu, Cr, and Zn) stress on photosynthesis, transpiration rate, and chlorophyll content in poplar hybrids, and observed a decrease in chlorophyll content, however, and an increase was also detected at higher heavy metals concentrations (200–500 mg/L). This fact may be explained by the biological accumulation of heavy metals that could be a factor to induced effects at high concentrations of heavy metals. BW9 soil elutriate sample could affect the photosynthetic process of *L. minor*, possibly due to PTE concentrations recorded [Al (0.222–0.365 mg/L), Mn (0.05–0.09 mg/L), Ni (0.007–0.011 mg/L), Zn (0.04–0.05 mg/L)] that could be incorporated in the chlorophyll molecule during the process. Indeed, the excess PTE accumulated could be used in this pigment biosynthesis, altering its functional property (Jmii and Dewez, 2021). Hou et al. (2007) already showed that total chlorophyll decreases in *L. minor* with the increase of PTE concentration (Cu > 10 mg/L; Cd > 0.5 mg/L), due to the peroxidation of chloroplast membranes mediated or replacing Mg in chlorophyll molecules by PTE. Radić et al. (2011) also suggested that Mg substitution in chlorophyll molecules by metal ions such as Cu, Zn, Cd, Hg, Pb, or Ni (recorded in the soil elutriates) could be the reason for the breakdown of photosynthesis in *L. minor*. Regarding the correlation analysis, only a positive and significant correlation between Ca (r = 0.403; *p* = 0.046) and total chlorophyll content was observed. Pal and Laloraya (1972) already suggest that calcium seems to exert an effect on chlorophyll formation through its control of the uptake of minerals essential for chlorophyll biosynthesis.

A significant increase in CAT activity was observed after exposure to the unburned soil elutriates from US1, US4, and UW5 (the most toxic samples regarding *L. minor* growth inhibition as well as for *D. magna* mortality – Figure 3; Table 1). These results could mean that these soil elutriates induced a significant increase in CAT activity, to counteract the oxidative stress caused by the elutriates. CAT belongs to the most important enzymes scavenging the reactive oxygen species in plant cells (Tuzet et al., 2019; Dvořák et al., 2021). CAT participates in the main defense system against the accumulation and toxicity of hydrogen peroxide, controlling the levels in cells, and converting them to water and oxygen (Msttés, 2000). CAT activity of *L. minor* exposed to PTE mainly displayed biphasic responses with increased metal concentration (Hou et al., 2007). Moreover, CAT activity increased when *L. minor* was exposed to soil elutriates with low pH, WHC, nutrient K, and trace metals Pb, Cu, and Cr values (Supplementary Table S1). Despite de low concentration of trace metals observed in the elutriates (Supplementary Table S1), Gwozdz et al. (1997) already reported that CAT increases in the presence of PTE low concentration. Indeed, a negative and significant correlation between CAT activity and WHC (*r* = −0.582; *p* = 0.002), K (*r* = −0.409; *p* = 0.042), and pH (*r* = −0.400; *p* = 0.048), while a positive and significant correlation was observed in OM (*r* = 0.460; *p* = 0.021).

The MDA content is an indicator of the cell damage endpoint and was determined to indicate the level of lipid peroxidation of *L. minor* fronds (Nunes et al., 2014). The results obtained indicate a significant increase in MDA contents after exposure to MBW6 and a significant decrease when it is exposed to MBW5 (Figure 3; burned coal wastes). After exposure to the MBW5 elutriate, *L. minor* response suggested that the antioxidative system could prevent oxidative damage (Foyer and Noctor, 2005; Varga et al., 2013). Indeed, MBW6 showed the highest concentration of F^-^ (0.08 mg/L), Al (0.962 mg/L), Cr (0.002 mg/L), and Cd (0.004 mg/L) than MBW5. MDA is a decomposition product of polyunsaturated fatty acids (PUFA) of biomembranes, and its increase shows that plants are under high-level antioxidant stress (Balen et al., 2011). The content of MDA in *L. minor* shows a positive and significant correlation between F^−^ (*r* = 0.428; *p* = 0.033), Al (*r* = 0.410; *p* = 0.042), Cd (*r* = 0.415; *p* = 0.039), and Cu (*r* = 0.398; *p* = 0.049). Radić et al. (2018) showed that Cd concentrations between 0.0016 and 0.003 mg/L increase the MDA concentration in *L. minor*, after exposure for 16 h to aqueous soil extracts from coal combustion. Cd is a non-essential metal and is known to interfere with plant growth and metabolic processes (Hou et al., 2007; Radić et al., 2013). Miras-Moreno et al. (2022) showed that Cu causes inhibition of plant growth, induces oxidative damage, and negatively affects photosynthetic activity, chlorophyll biosynthesis, and plant mineral nutrition at up to 10 mg/L. Other studies with Ni and Zn showed that these metals induce cell division and hence plant growth, causing genotoxicity and oxidative damage effects (e.g., Rout et al., 2001).

## 4 Conclusion

The present research showed that the Fojo coal mining wastes present adverse effects on aquatic species under study. The results demonstrated that: i) physical and chemical characteristics of soil elutriates showed low contents of bioavailable PTE (e.g., Al, Pb, Cd, Ni, Zn) where the most responsible compounds for the toxicity of the elutriates, seem to be Fe and Al; ii) all the Fojo soil elutriates affect the bioluminescence of *A. fischeri* (most sensitive species); iii) soils without mining influence (US), unburned coal waste (UW), and mixed burnt and unburnt coal wastes (MBW) were the most toxic for *L. minor* (affecting the growth inhibition and biochemical parameters); iv) *D. magna* was the less sensitive species, being only drastically affected by two soil elutriates (US1 and UW5, that caused 100% mortality).

The here-presented results showed that the bioavailable of several compounds (metals) present in coal mining wastes could reach the aquatic ecosystems mainly by mobilization and percolation, inducing significant effects in aquatic species, namely, at the base of the trophic chain (bacteria). Nevertheless, further studies will be needed to assess the toxicity effects of coal waste areas as well as surrounding ecosystems, in different species, exposure periods, and sensitive endpoints. This ecotoxicological approach will provide realistic data that will allow the proposal of management measures and minimization of environmental risk in these areas.

## Data Availability

The original contributions presented in the study are included in the article/Supplementary Material, further inquiries can be directed to the corresponding author.
