# Supplementary material for: Assessment of ecotoxicological effects of Fojo coal mine waste elutriate in aquatic species (Douro Coalfield, North Portugal)
Source: Front Toxicol. 2024 Feb 23;6:1334169. doi: 10.3389/ftox.2024.1334169 (PMC10920227; doi:10.3389/ftox.2024.1334169)
Supplement: Supplementary file 1 [file Table1.pdf]

Table S1. Results of physical and chemical parameters (for parameters abbreviations see 2.2 section) measured in the soil elutriates.

Samples	pH	Cond	WHC	OM	HCO_3	Cl	SO_4	F^-	NO_3	NO_2	NH_4	Na	K	Ca	Mg	Fe	Cu	Zn	Al	Mn	As	Cd	Ni	Cr	Pb
		μS/cm	%	%	mg/L														μg/L						
**US1**	4.3	59.2	10.1	9.0	2.52	0.74	0.90	0.01	0.52	0.00	0.22	0.81	0.52	0.79	0.21	67.62	0.00	0.00	39.24	2.70	0.50	0.00	1.45	0.85	1.00
**US4**	4.4	53.3	10.2	9.3	2.02	0.85	0.44	0.01	0.43	0.00	0.16	1.07	0.49	0.53	0.15	44.49	0.00	0.00	33.69	0.50	0.35	0.00	1.30	0.00	0.00
**US5**	4.5	42.7	14.5	7.2	3.28	1.31	0.95	0.01	0.70	0.01	0.16	1.73	0.52	0.46	0.15	63.44	0.00	0.00	59.68	0.00	1.85	0.00	0.10	0.10	0.00
**UW1**	4.8	147.6	27.1	16.2	0.69	0.34	12.83	0.01	0.88	0.01	0.04	0.39	0.78	2.68	1.30	20.70	0.00	0.03	8.10	118.30	0.00	0.00	11.60	0.00	1.60
**UW3**	5.1	155.0	26.8	19.9	2.33	0.84	15.92	0.03	0.54	0.01	0.00	0.49	1.06	4.44	1.74	79.66	0.00	0.03	49.53	160.70	1.15	0.00	4.70	0.00	0.00
**UW5**	3.5	321.7	23.0	19.7	0.00	0.34	17.15	0.01	0.31	0.00	0.04	0.25	0.51	0.87	0.54	82.59	0.01	0.03	199.30	51.30	0.00	0.00	3.70	0.00	1.70
**MBW1**	3.8	208.3	23.9	8.3	0.00	0.47	15.94	0.03	0.51	0.00	0.03	0.95	0.81	1.00	0.72	45.66	0.01	0.02	617.22	27.90	0.20	0.20	6.85	1.25	3.40
**MBW2**	4.1	255.0	23.9	11.9	0.00	0.27	20.14	0.03	0.68	0.00	0.20	1.04	0.57	2.64	1.59	27.80	0.01	0.01	300.86	69.35	0.55	0.00	8.80	0.65	4.00
**MBW3**	4.0	238.7	23.9	8.9	0.32	0.42	20.39	0.02	0.80	0.00	0.34	1.20	0.68	18.33	1.74	25.78	0.00	0.00	187.56	77.25	0.00	0.20	9.90	0.00	3.50
**MBW4**	4.2	107.7	28.0	10.4	0.00	0.28	10.79	0.04	0.23	0.00	0.01	0.58	0.93	1.49	0.56	0.00	0.00	0.00	171.00	20.70	0.60	0.00	6.80	0.00	2.95
**MBW5**	4.0	202.3	24.9	10.4	0.00	0.33	17.47	0.04	0.56	0.00	0.02	0.75	0.85	2.11	0.98	20.67	0.01	0.00	412.11	56.40	0.00	0.10	8.45	0.00	2.70
**MBW6**	3.9	269.7	23.8	10.0	0.00	0.30	32.23	0.08	0.72	0.00	0.06	0.81	0.80	4.56	1.96	10.97	0.02	0.02	962.50	122.90	0.00	0.40	17.50	0.25	0.00
**BW3**	4.1	179.3	30.7	7.2	0.00	0.34	17.85	0.03	0.54	0.00	0.12	0.69	0.70	2.62	1.24	32.45	0.00	0.04	365.50	50.80	0.00	0.00	11.45	0.00	0.00
**BW4**	4.3	103.3	29.3	10.2	0.00	0.36	11.08	0.03	0.63	0.00	0.00	0.61	0.75	1.51	0.77	22.03	0.00	0.02	165.60	44.30	0.75	0.00	5.45	0.00	0.00
**BW8**	4.2	211.3	31.4	6.7	0.00	0.34	18.93	0.03	0.45	0.00	0.00	0.91	0.81	3.25	1.22	27.13	0.00	0.05	323.15	72.20	0.60	0.00	7.95	0.00	0.00
**BW9**	4.1	208.7	24.8	4.3	0.00	0.28	21.06	0.04	0.28	0.00	0.01	0.77	0.74	3.88	1.67	27.04	0.00	0.05	221.90	99.15	0.25	0.00	7.95	0.00	0.00
**BW10**	4.6	100.6	35.8	4.5	0.00	0.37	8.98	0.01	0.22	0.00	0.00	0.57	1.16	1.81	0.61	77.28	0.00	0.13	29.63	78.70	0.25	0.00	3.83	0.00	0.00
**CL3**	4.8	75.2	42.7	3.6	2.21	0.57	4.86	0.01	1.00	0.00	0.03	0.80	0.55	0.92	0.64	100.68	0.00	0.00	9.19	38.00	0.15	0.00	23.75	0.00	0.95
**CL4**	5.1	32.6	46.3	6.2	2.02	0.73	1.23	0.01	0.79	0.01	0.06	0.71	0.51	0.54	0.25	178.58	0.00	0.00	2.23	5.85	0.00	0.00	0.65	0.00	1.60
**CL8**	4.8	56.2	39.6	3.9	1.51	0.48	3.53	0.01	0.43	0.00	0.03	0.55	0.57	0.87	0.53	60.10	0.00	0.00	11.50	45.40	0.00	0.00	0.95	0.00	0.00
**CL9**	4.6	142.0	34.9	6.6	0.00	0.60	11.82	0.02	1.19	0.01	161.0	0.84	0.56	2.28	1.13	45.79	0.00	0.00	130.15	101.15	0.25	0.00	5.95	0.00	0.00
**CL10**	5.0	45.7	41.6	5.3	3.15	0.49	3.12	0.01	0.89	0.00	0.11	0.57	0.77	0.93	0.37	58.80	0.00	0.02	12.14	21.50	0.40	0.00	2.10	0.00	0.20
**DS1**	4.1	168.0	35.2	5.5	0.95	0.62	10.71	0.01	0.58	0.00	0.16	0.95	0.71	1.56	0.70	59.88	0.01	0.01	171.14	136.30	0.80	0.00	0.10	0.70	0.40
**DS3**	4.2	366.0	36.2	5.7	0.00	0.70	33.18	0.03	0.22	0.00	0.33	1.69	1.12	3.36	4.32	36.08	0.00	0.02	342.96	368.90	0.90	0.20	17.10	0.00	0.00
**DS5**	4.8	68.2	36.7	4.8	2.14	0.54	4.74	0.01	1.48	0.01	0.11	0.67	0.83	1.03	0.63	19.19	0.00	0.00	14.02	350.75	0.00	0.00	3.60	0.00	0.00
